# Association between cumulative changes of the C-reactive protein-triglyceride glucose index and the incidence of rapid kidney function decline: a nationwide prospective cohort study

**DOI:** 10.3389/fnut.2026.1795444

**Published:** 2026-04-13

**Authors:** Lina Huang, Tingting Su, Tianbao Liao, Yang Lu, Lu-Huai Feng

**Affiliations:** 1 Department of Clinical Nutrition, Guangxi Medical University Cancer Hospital, Nanning, China; 2 Department of ECG Diagnostics, the People’s Hospital of Guangxi Zhuang Autonomous Region, Nanning, China; 3 Department of President’s Office, Youjiang Medical University for Nationalities, Baise, China; 4 Department of International Medical, Guangxi Medical University Cancer Hospital, Nanning, China; 5 Department of Endocrinology and Metabolism Nephrology, Guangxi Medical University Cancer Hospital, Nanning, China

**Keywords:** chronic kidney disease progression, C-reactive protein–triglyceride glucose index, cumulative metabolic inflammation, kidney function decline, population-based cohort

## Abstract

**Background:**

Rapid kidney function decline (RKFD) is an early indicator of chronic kidney disease and a precursor to kidney failure, yet early detection is challenging. Inflammation and metabolic imbalance may cause kidney damage, and the C-reactive protein–triglyceride glucose index (CTI) represents both factors. This study aims to explore the associations of cumulative changes of CTI (cuCTI) with RKFD risk in middle-aged and older adults.

**Methods:**

We analyzed data from 6,888 individuals aged 45 and older from the 2011 and 2015 China Health and Retirement Longitudinal Study (CHARLS). Using multivariate logistic regression models, we explored the association between cuCTI, CTI control levels, and RKFD risk. We also used restricted cubic spline (RCS) models for dose-response patterns and conducted subgroup analyses based on sex, education, smoking, alcohol use, hypertension, diabetes, dyslipidemia, and CKD stage. Sensitivity analyses were performed on original, imputed, and pooled datasets to ensure robust results. A supplementary cross-sectional analysis using National Health and Nutrition Examination Survey analysis (NHANES) data examined the association between CTI and eGFR using weighted linear regression and RCS models.

**Results:**

Over a four-year period, 262 participants (3.8%) developed RKFD. Increased cuCTI and poor CTI control were linked to a higher RKFD risk, with each one-unit cuCTI increase raising the odds by 18% (OR = 1.18, 95% CI: 1.13–1.22). Those with consistently high or rising CTI faced the greatest risk. The dose-response curve indicated a linear increase in RKFD risk with higher cuCTI, and subgroup analyses showed consistent results across all groups. Sensitivity analyses confirmed the stability of these associations across datasets. Similar associations were observed in the supplementary analysis using NHANES data, with higher CTI levels associated with lower eGFR.

**Conclusion:**

Higher cumulative CTI levels were associated with an increased risk of rapid kidney function decline, and the association was most pronounced among individuals with persistently high CTI levels. Monitoring long-term inflammatory and metabolic status may help identify individuals at risk of renal function decline in middle-aged and older adults.

## Introduction

Chronic kidney disease (CKD) represents a significant global health challenge, affecting millions worldwide and contributing to elevated morbidity and mortality rates (1–3). Rapid kidney function decline (RKFD) constitutes an early phase in the progression of CKD, often manifesting prior to the clinical recognition of CKD and serving as a strong predictor of end-stage kidney disease and cardiovascular events (4). Despite its clinical significance, early detection of RKFD remains challenging. Conventional biomarkers, such as serum creatinine and cystatin C, typically indicate renal damage only after substantial functional impairment has occurred (5). Moreover, these markers are influenced by variables such as age, muscle mass, and metabolic status, thereby limiting their diagnostic precision. Currently, there is an absence of a straightforward and reliable biomarker capable of identifying early and subtle alterations in renal health. Consequently, there is an urgent need to identify sensitive and dynamic indicators that can detect early metabolic or inflammatory changes preceding detectable kidney damage.

Chronic inflammation and metabolic imbalance are intricately associated with renal injury. Inflammatory processes can lead to damage of blood vessels and glomeruli, while insulin resistance and lipid accumulation can exacerbate oxidative stress and promote fibrosis (6–8). There are multiple interactions between inflammation and metabolic disorders, and these two processes often occur simultaneously, potentially accelerating the deterioration of kidney function. C-reactive protein (CRP) is widely utilized as a marker of systemic inflammation, whereas the triglyceride–glucose (TyG) index serves as a straightforward and reliable indicator of insulin resistance. Each of these markers reflects a distinct aspect of the inflammatory–metabolic state (9). A combined evaluation of these markers may offer a more comprehensive understanding of chronic inflammatory and metabolic stress. The C-reactive protein–triglyceride glucose index (CTI) was formulated to consolidate CRP and TyG into a unified metric, thereby encapsulating both inflammatory and metabolic activities (10). Although CTI has been linked with metabolic and cardiovascular conditions (11, 12), its implications for renal deterioration remain unclear. Considering the dynamic nature of inflammation and metabolism, a solitary measurement may not sufficiently capture their long-term effects. The cumulative changes of the CTI (cuCTI) account for both the magnitude and duration of exposure (13), potentially offering a more accurate reflection of the chronic inflammatory and metabolic burden that impacts renal health.

While inflammation and metabolic imbalance are established contributors to renal damage, the extent to which their combined long-term effects may serve as indicators of early renal decline remains unclear. Previous research has predominantly concentrated on isolated measurements of inflammatory or metabolic markers (14–16), potentially failing to capture the cumulative exposure that drives renal deterioration. There is a paucity of evidence from large, community-based cohorts, particularly among middle-aged and older adults who are at elevated risk. Investigating a cumulative measure that encapsulates both inflammatory and metabolic load over time could enhance the early identification of individuals at risk. This study aimed to investigate the association between cuCTI and the risk of RKFD in middle-aged and older adults.

## Materials and methods

### Study design and data source

This study was a population-based prospective cohort study using data from the CHARLS (17).^1^ CHARLS is a national survey of Chinese adults aged 45 years and older. It was launched in 2011 by the National School of Development at Peking University. Participants were selected through a multistage probability sampling process to represent the national population. Baseline data were collected in 2011. Follow-up surveys were carried out every 2–3 years through structured interviews, physical examinations, and laboratory tests. In addition, supplementary cross-sectional analyses were conducted using data from the National Health and Nutrition Examination Survey (NHANES). NHANES is a nationally representative survey of the civilian, non-institutionalized population in the United States and employs a complex, multistage probability sampling design (18).

### Participants

**FIGURE 1:**
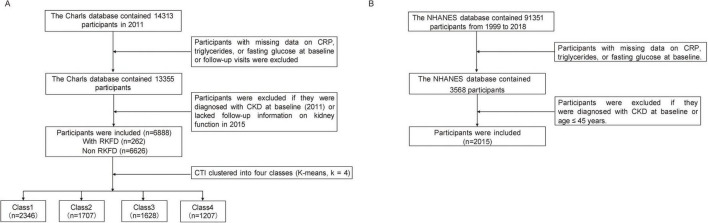
Flowchart of participant selection for the CHARLS and NHANES analyses. **(A)** Participant selection from CHARLS for the prospective analysis of cuCTI, CTI control patterns, and RKFD. **(B)** Participant selection from NHANES for the supplementary cross-sectional analysis of the association between CTI and kidney function.

This study was primarily based on data from the CHARLS, a nationally representative cohort of Chinese adults aged 45 years and older. Participants who completed both the 2011 baseline and the 2015 follow-up surveys were eligible for inclusion. We included individuals with available measurements of CRP, triglycerides (TG), and fasting blood glucose (FBG) at both time points. These variables were used to calculate the CTI and cuCTI. Participants were excluded if they had CKD at baseline or if follow-up data on kidney function were unavailable in 2015. The detailed participant selection process is shown in Figure 1A. In the CHARLS study, venous blood samples were collected during the biomarker surveys conducted in 2011 and repeated in 2015. These two waves represent the only survey cycles in which comprehensive laboratory measurements, including CRP, TG, and FBG, were simultaneously available for the same participants in the publicly released CHARLS dataset. Therefore, CTI was calculated at both time points, and cuCTI was derived from these repeated measurements to reflect long-term metabolic–inflammatory exposure.

For the supplementary analysis using NHANES data, participants aged 45 years or older from the NHANES cycles conducted between 1999 and 2018 were included. Individuals with available measurements of CRP, triglycerides, fasting glucose, and serum creatinine were eligible for inclusion, as these variables were required to calculate CTI and assess kidney function. Participants with CKD at baseline were excluded to ensure consistency with the inclusion criteria applied in the CHARLS cohort. The detailed participant selection process is shown in Figure 1B.

### Data extraction

The primarily data were collected from the 2011 and 2015 waves of the CHARLS cohort. Trained investigators gathered all information through standardized questionnaires, physical measurements, and laboratory tests. Demographic and lifestyle data included age, gender, body mass index (BMI), education level, smoking status, and drinking status. Chronic disease history covered hypertension, chronic kidney disease, diabetes, dyslipidemia, stroke, heart disease, chronic lung disease, liver disease, and digestive disease. Laboratory data were obtained from fasting blood samples and included FBG, TG, total cholesterol (TC), high-density lipoprotein cholesterol (HDL-C), low-density lipoprotein cholesterol (LDL-C), hemoglobin A1c (HbA1c), serum creatinine (Scr), blood urea nitrogen (BUN), uric acid, hemoglobin, and CRP.

For the supplementary analysis using NHANES data, demographic variables (age and sex), clinical characteristics (body mass index, smoking status, alcohol consumption, hypertension, and diabetes), and laboratory measurements (CRP, triglycerides, fasting glucose, and serum creatinine) were extracted from the corresponding NHANES.

### Definitions and outcomes

#### Calculation of CTI and cuCTI

The cuCTI is a composite indicator reflecting cumulative inflammatory–metabolic burden over time by integrating repeated measurements of CRP, triglycerides, and FBG. Both CTI and cuCTI are dimensionless indices. First, the CTI was calculated for each participant at both survey waves using the following formula: CTI = 0.412 × ln (CRP [mg/L]) + ln [Triglyceride (mg/dL) × FBG (mg/dL)/2]. The weighting coefficient for CRP (0.412) was adopted from previously published studies in which the CTI index and its calculation formula were originally reported (19, 20). The cuCTI was then calculated to represent the cumulative exposure to CTI as follows (21): cuCTI = [(CTI_2011_ + CTI_2015_)/2] × (2015 - 2011). CTI_2011_ and CTI_2015_ represent CTI values obtained from the 2011 (Wave 1) and 2015 (Wave 3) CHARLS surveys. This approach for calculating cuCTI has been applied in previous longitudinal studies (21–23).

#### Assessment of RKFD

The estimated glomerular filtration rate (eGFR) was determined using the combined creatinine–cystatin C equation (24), which has been demonstrated to offer superior accuracy compared to equations utilizing either marker independently. RKFD was defined as an annualized decline in eGFR of ≥ 5 mL/min/1.73 m^2^, consistent with the KDIGO 2012 guideline for rapid CKD progression. This threshold has been widely adopted in longitudinal epidemiological studies as a clinically meaningful indicator of accelerated kidney function loss (25–27). The annualized change in eGFR was calculated by subtracting the baseline eGFR_2011_ from the eGFR at the conclusion of the follow-up period (2015) and dividing by the total duration of follow-up in years: (eGFR_2015_ – eGFR_2011_)/follow-up years. The mean follow-up duration was 4 years. Participants exhibiting an annualized eGFR decline of ≥ 5 mL/min/1.73 m^2^ were classified as experiencing RKFD.

#### Missing data handling

For the CHARLS cohort, missing data were presumed to be missing at random (MAR). Prior to conducting analyses, we assessed the extent of missing data across all study variables. The proportion of missing values varied from 0 to 12.6%, with eGFR and body mass index (BMI) exhibiting the highest rates of missing data at 12.6 and 7.0%, respectively. Most other clinical and laboratory variables demonstrated missing rates below 3%. Variables such as gender, education, TC, TG, LDL-C, HDL-C, cuCTI, and CTI were complete.

To address missing data and reduce potential bias, we employed multiple imputation by chained equations (MICE) (28). Five imputed datasets (*m* = 5) were generated, and each dataset was analyzed independently. For continuous variables, predictive mean matching was utilized, while logistic regression models were applied to binary variables. All variables included in the regression models were incorporated into the imputation process to ensure consistency between the imputation and analysis models. Regression analyses were performed separately within each imputed dataset, and the resulting estimates were subsequently combined using Rubin’s rules to obtain pooled results. This approach is consistent with standard multiple imputation procedures and allows proper accounting for the uncertainty introduced by missing data. These pooled estimates were subsequently used for all statistical analyses.

For the NHANES cohort, participants with missing values in key variables (CRP, triglycerides, fasting glucose, or serum creatinine) were excluded, and analyses were performed using complete-case data. This approach was adopted because these measurements were required to calculate CTI and assess kidney function, and imputing key exposure or outcome variables may introduce model-dependent bias (29). Similar handling of missing key variables has been commonly applied in previous NHANES-based studies (30, 31).

### Statistical analysis

Continuous variables with skewed distributions were expressed as medians with interquartile ranges. Categorical variables were described using frequencies and their associated percentages. The Mann–Whitney U test was applied for intergroup comparisons of continuous variables, whereas the chi-square test was used for analyzing categorical variables.

In the CHARLS cohort, K-means clustering was employed to categorize participants into four distinct CTI control levels, based on their CTI values from the years 2011 and 2015. This unsupervised learning technique partitions N observations into K clusters. Each participant is assigned to the cluster with the nearest median CTI value. The algorithm aims to minimize the within-cluster sum of squares, thereby capturing diverse long-term CTI control patterns over time. The optimal number of clusters was identified using the elbow method, which evaluates the reduction in total within-cluster variance as K increases. In this approach, the “elbow” represents the point at which further increases in the number of clusters yield only marginal reductions in within-cluster variance. As illustrated in Supplementary Figure 1A, the rate of decline in within-cluster variance became noticeably smaller after *K* = 4. Considering both the elbow criterion and the interpretability of longitudinal CTI patterns, four clusters were selected to characterize distinct CTI trajectories. Consequently, participants were classified into four CTI control levels according to the nearest cluster center (Supplementary Figure 1B). These four levels encapsulated distinct metabolic patterns over the follow-up period: Class 1 exhibited stable and low CTI values, Class 2 demonstrated a steady increase, Class 3 showed a decline, and Class 4 maintained persistently high levels (Supplementary Figure 1C). The median CTI values for each class at both time points were utilized to describe the overall trend.

Multivariable logistic regression was performed to examine the associations of cuCTI and CTI control levels with RKFD. Three models were constructed for stepwise adjustment: Model 1 was unadjusted; Model 2 was adjusted for age, gender, BMI, and education level; and Model 3 was further adjusted for lifestyle factors, comorbidities, and laboratory variables, including smoking status, alcohol consumption, hypertension, diabetes, dyslipidemia, cardiovascular disease, chronic lung disease, liver disease, digestive disease, total cholesterol, HDL-C, LDL-C, HbA1c, uric acid, and hemoglobin. Adjusted odds ratios (ORs) and 95% confidence intervals (CIs) were derived from pooled estimates using Rubin’s rules following multiple imputation. Sensitivity analyses were conducted using Model 3 across the original, imputed, and pooled datasets to assess the robustness of the associations.

A restricted cubic spline regression model with three knots was employed to investigate the dose–response relationship between cuCTI and the risk of RKFD. Subgroup analyses were conducted based on variables such as hypertension, diabetes, lung disease, heart disease, stroke, dyslipidemia, liver disease, digestive disease, drinking status, smoking status, gender, and education level. Interaction terms were incorporated to assess potential differences in associations across these subgroups.

To evaluate potential reverse causality, we conducted an inverse analysis to examine whether baseline renal function predicted subsequent changes in CTI. Changes in CTI were defined as the difference between CTI values measured in 2015 and 2011. Multivariable linear regression models were fitted with adjustment for age, sex, body mass index, education level, smoking status, alcohol consumption, hypertension, diabetes, cardiovascular disease (including heart disease and stroke), chronic lung disease, liver disease, digestive disease, HbA1c, and hemoglobin levels.

For the supplementary analysis in NHANES cohort, weighted linear regression models were used to evaluate the association between CTI and eGFR. Restricted cubic spline analysis was further applied to explore the potential non-linear relationship between CTI and kidney function. These analyses were conducted to provide complementary population-level evidence supporting the findings from the CHARLS cohort.

All analyses were conducted using R software (version 4.3.0; R Foundation for Statistical Computing, Vienna, Austria). Two-sided *P* values < 0.05 were considered statistically significant.

## Results

### Characteristics of the study population

A total of 6,888 participants were included in the analysis from an initial cohort of 14,313 individuals screened nationally (refer to Figure 1). During the follow-up period, 262 participants (3.8%) developed RKFD. Table 1 presents the characteristics of the study population at the conclusion of the follow-up in 2015, stratified by RKFD status. Participants with RKFD were slightly older than those without RKFD and had a higher prevalence of hypertension, diabetes, stroke, and dyslipidemia. The prevalence of other health conditions, such as cardiovascular, pulmonary, or hepatic diseases, did not significantly differ between the groups. Lifestyle factors, including smoking, alcohol consumption, and educational attainment, were comparable across both cohorts. However, individuals with RKFD demonstrated poorer metabolic and renal profiles, characterized by elevated BMI, blood glucose levels, triglycerides, HbA1c, uric acid, and serum creatinine, alongside reduced HDL-C and eGFR. Additionally, both the cuCTI and the CTI in 2015 were elevated in the RKFD group.

**TABLE 1:** The characteristics of the study participants at the end of follow-up (2015).

Variable	Total (*n* = 6,888)	Non-RKFD (*n* = 6,626)	RKFD (*n* = 262)	*P-*value
Age, years	62 (56, 69)	62 (56, 69)	63 (57, 70)	0.047
Gender, n (%)				
Female	3785 (55.0)	3640 (54.9)	145 (55.3)	0.947
Male	3103 (45.0)	2986 (45.1)	117 (44.7)	
Hypertension, n (%)				
No	4368 (63.4)	4233 (63.9)	135 (51.5)	< 0.001
Yes	2520 (36.6)	2393 (36.1)	127 (48.5)	
Diabetes, n (%)				
No	6170 (89.6)	5947 (89.8)	223 (85.1)	0.021
Yes	718 (10.4)	679 (10.2)	39 (14.9)	
Lung disease, n (%)				
No	5902 (85.7)	5674 (85.6)	228 (87.0)	0.589
Yes	986 (14.3)	952 (14.4)	34 (13.0)	
Heart disease, n (%)				
No	5599 (81.3)	5387 (81.3)	212 (80.9)	0.939
Yes	1289 (18.7)	1239 (18.7)	50 (19.1)	
Stroke, n (%)				
No	6622 (96.1)	6379 (96.3)	243 (92.7)	0.006
Yes	266 (3.9)	247 (3.7)	19 (7.3)	
Dyslipidemia, n (%)				
No	5461 (79.3)	5288 (79.8)	173 (66.0)	< 0.001
Yes	1427 (20.7)	1338 (20.2)	89 (34.0)	
Liver disease, n (%)				
No	6445 (93.6)	6202 (93.6)	243 (92.7)	0.672
Yes	443 (6.4)	424 (6.4)	19 (7.3)	
CKD, n (%)				
No	6536 (94.9)	6292 (95.0)	244 (93.1)	0.240
Yes	352 (5.1)	334 (5.0)	18 (6.9)	
Digeste disease, n (%)				
No	4736 (68.8)	4562 (68.8)	174 (66.4)	0.443
Yes	2152 (31.2)	2064 (31.2)	88 (33.6)	
Drinking status, n (%)				
Never	3768 (54.7)	3623 (54.7)	145 (55.3)	0.882
Ever	3120 (45.3)	3003 (45.3)	117 (44.7)	
Smoking status, n (%)				
Non-smokers	3945 (57.3)	3783 (57.1)	162 (61.8)	0.145
Smokers	2943 (42.7)	2843 (42.9)	100 (38.2)	
Education, n (%)				
Lower than high school	4839 (70.3)	4650 (70.2)	189 (72.1)	0.541
High school or above	2049 (29.7)	1976 (29.8)	73 (27.9)	
BMI, kg/m^2^	23.6 (21.2, 26.2)	23.6 (21.2, 26.1)	24.4 (21.8, 27.0)	0.002
BUN, mg/dL	15.1 (12.6, 18.2)	14.8 (12.3, 18.2)	16.4 (13.4, 20.2)	< 0.001
Glucose, mg/dL	95.5 (88.3, 106.3)	95.5 (88.3, 106.3)	99.1 (91.9, 113.5)	< 0.001
TC, mg/dL	182.2 (159.8, 206.2)	182.0 (159.8, 206.2)	185.3 (161.0, 213.9)	0.216
TG, mg/dL	116.8 (83.2, 170.8)	115.9 (83.2, 169.9)	145.1 (99.1, 229.2)	< 0.001
HDL-C, mg/dL	49.8 (43.2, 57.5)	49.8 (43.2, 57.5)	45.6 (40.2, 54.1)	< 0.001
LDL-C, mg/dL	101.2 (82.6, 119.7)	101.2 (82.6, 119.7)	101.7 (81.9, 122.8)	0.661
HbA1c, %	5.8 (5.5, 6.2)	5.8 (5.5, 6.2)	5.9 (5.6, 6.5)	< 0.001
Uric acid, mg/dL	4.8 (3.9, 5.7)	4.8 (3.9, 5.7)	5.4 (4.5, 6.8)	< 0.001
Hemoglobin, g/dL	13.6 (12.5, 14.7)	13.6 (12.5, 14.7)	13.4 (12.2, 14.6)	0.076
Scr, mol/L	0.8 (0.7, 0.9)	0.8 (0.7, 0.9)	1.0 (0.8, 1.4)	< 0.001
eGFR, mL/min/1.73 m	97.8 (85.2, 109.2)	98.5 (86.3, 109.6)	73.8 (63.7, 89.6)	< 0.001
cuCTI	35.0 (33.2, 37.2)	35.0 (33.1, 37.1)	36.7 (34.3, 39.4)	< 0.001
CTI	8.8 (8.3, 9.4)	8.8 (8.3, 9.4)	9.2 (8.6, 10.0)	0.085

Table 2 provides a summary of the participant characteristics, categorized into four CTI control classes via k-means clustering. Class 1 (*n* = 2346) exhibited consistently low and stable CTI values over time, maintaining an average of approximately 8.0 in both 2011 and 2015. Class 2 (*n* = 1707) demonstrated a progressive increase in CTI, rising from 8.5 (95% CI: 8.2, 8.7) in 2011 to 9.2 (95% CI: 9.0, 9.6) in 2015. Class 3 (n = 1628) initially presented with higher CTI values but experienced a decline from 9.2 (95% CI: 9.0, 9.5) to 8.8 (95% CI: 8.5, 9.1). Class 4 (*n* = 1207) had the highest CTI levels, with a slight increase from 9.8 (95% CI: 9.4, 10.3) to 10.1 (95% CI: 9.7, 10.5) (refer to Supplementary Figure 1C). Notably, clinical and metabolic characteristics varied among the four classes. Participants in Classes 2 and 4, characterized by rising or persistently high CTI, exhibited higher prevalence rates of hypertension, diabetes, dyslipidemia, and heart disease. These individuals also had elevated BMI, blood glucose, triglycerides, HbA1c, and uric acid levels, alongside reduced HDL-C and eGFR. Conversely, individuals in Class 1 displayed the most favorable metabolic and renal profiles.

**TABLE 2:** The characteristics according to CTI control level classes.

Variable	Class 1 (*n* = 2,346)	Class 2 (*n* = 1,707)	Class 3 (*n* = 1,628)	Class 4 (*n* = 1,207)	*P-*value
Age, years	62 (55, 69)	62 (55, 69)	62 (56, 69)	63 (56, 68)	0.138
Gender, n (%)					
Female	1170 (49.9)	975 (57.1)	917 (56.3)	723 (59.9)	< 0.001
Male	1176 (50.1)	732 (42.9)	711 (43.7)	484 (40.1)	
RKFD					
No	2296 (97.9)	1645 (96.4)	1576 (96.8)	1109 (91.9)	< 0.001
Yes	50 (2.1)	62 (3.6)	52 (3.2)	98 (8.1)	
Hypertension, n (%)					
No	1748 (74.5)	1072 (62.8)	993 (61.0)	555 (46.0)	< 0.001
Yes	598 (25.5)	635 (37.2)	635 (39.0)	652 (54.0)	
Diabetes, n (%)					
No	2247 (95.8)	1563 (91.6)	1463 (89.9)	897 (74.3)	< 0.001
Yes	99 (4.2)	144 (8.4)	165 (10.1)	310 (25.7)	
Lung disease, n (%)					
No	2041 (87.0)	1448 (84.8)	1377 (84.6)	1036 (85.8)	0.113
Yes	305 (13.0)	259 (15.2)	251 (15.4)	171 (14.2)	
Heart disease, n (%)					
No	1993 (85.0)	1395 (81.7)	1303 (80.0)	908 (75.2)	< 0.001
Yes	353 (15.0)	312 (18.3)	325 (20.0)	299 (24.8)	
Stroke, n (%)					
No	2277 (97.1)	1648 (96.5)	1572 (96.6)	1125 (93.2)	< 0.001
Yes	69 (2.9)	59 (3.5)	56 (3.4)	82 (6.8)	
Dyslipidemia, n (%)					
No	2073 (88.4)	1373 (80.4)	1241 (76.2)	774 (64.1)	< 0.001
Yes	273 (11.6)	334 (19.6)	387 (23.8)	433 (35.9)	
Liver disease, n (%)					
No	2192 (93.4)	1613 (94.5)	1521 (93.4)	1119 (92.7)	0.258
Yes	154 (6.6)	94 (5.5)	107 (6.6)	88 (7.3)	
CKD, n (%)					
No	2235 (95.3)	1613 (94.5)	1540 (94.6)	1148 (95.1)	0.645
Yes	111 (4.7)	94 (5.5)	88 (5.4)	59 (4.9)	
Digeste disease, n (%)					
No	1539 (65.6)	1171 (68.6)	1166 (71.6)	860 (71.3)	< 0.001
Yes	807 (34.4)	536 (31.4)	462 (28.4)	347 (28.7)	
Drinking status, n (%)					
Never	1221 (52.0)	959 (56.2)	893 (54.9)	695 (57.6)	0.007
Ever	1125 (48.0)	748 (43.8)	735 (45.1)	512 (42.4)	
Smoking status, n (%)					
Non-smokers	1287 (54.9)	1001 (58.6)	929 (57.1)	728 (60.3)	0.009
Smokers	1059 (45.1)	706 (41.4)	699 (42.9)	479 (39.7)	
Education, n (%)					
Lower than high school	1639 (69.9)	1236 (72.4)	1123 (69.0)	841 (69.7)	0.144
High school or above	707 (30.1)	471 (27.6)	505 (31.0)	366 (30.3)	
BMI, kg/m^2^	22.1 (20.1, 24.3)	24.0 (21.7, 26.3)	24.0 (21.7, 26.6)	25.6 (23.4, 28.1)	< 0.001
BUN, mg/dL	15.4 (12.6, 18.5)	15.1 (12.3, 18.5)	14.8 (12.5, 18.2)	14.8 (12.3, 17.9)	0.033
Glucose, mg/dL	90.1 (84.7, 97.3)	99.1 (91.9, 109.9)	95.5 (88.3, 103.6)	109.9 (97.3, 144.1)	< 0.001
TC, mg/dL	173.7 (154.4, 195.4)	185.7 (163.7, 210.4)	182.6 (159.8, 206.2)	196.5 (171.4, 223.2)	< 0.001
TG, mg/dL	80.5 (65.5, 100.0)	150.4 (113.3, 196.5)	115.0 (89.4, 146.0)	235.4 (171.7, 336.3)	< 0.001
HDL-C, mg/dL	54.4 (47.5, 62.5)	47.9 (42.1, 55.2)	49.8 (43.6, 56.8)	43.6 (39.0, 49.8)	< 0.001
LDL-C, mg/dL	97.7 (81.1, 114.7)	103.9 (84.6, 123.6)	105.8 (86.5, 123.2)	98.5 (79.2, 120.5)	< 0.001
HbA1c, %	5.7 (5.5, 6.0)	5.8 (5.5, 6.1)	5.8 (5.6, 6.1)	6.2 (5.8, 7.1)	< 0.001
Uric acid, mg/dL	4.4 (3.7, 5.3)	4.9 (4.0, 5.8)	4.8 (4.0, 5.8)	5.3 (4.4, 6.3)	< 0.001
Hemoglobin, g/dL	13.4 (12.4, 14.5)	13.6 (12.6, 14.8)	13.6 (12.5, 14.8)	13.8 (12.7, 15.0)	< 0.001
Scr, mol/L	0.8 (0.7, 0.9)	0.8 (0.7, 0.9)	0.8 (0.7, 0.9)	0.7 (0.6, 0.9)	0.045
eGFR, mL/min/1.73 m	100.5 (87.4, 110.6)	96.8 (84.6, 109.0)	97.3 (84.7, 108.1)	94.9 (82.2, 107.7)	< 0.001
cuCTI	32.4 (31.4, 33.2)	35.3 (34.5, 36.4)	36.0 (35.0, 37.0)	39.7 (38.8, 41.2)	< 0.001
CTI_2011_	8.1 (7.8, 8.4)	8.5 (8.2, 8.7)	9.2 (9.0, 9.5)	9.8 (9.4, 10.3)	< 0.001
CTI_2015_	8.2 (7.9, 8.3)	9.2 (9.0, 9.6)	8.8 (8.5, 9.1)	10.1 (9.7, 10.5)	< 0.001

### Association between cuCTI, CTI control levels and RKFD incidence

Table 3 presents the logistic regression results for CTI control levels, cuCTI, and RKFD. Consistent patterns were observed across all models. Both higher cuCTI and unfavorable CTI control levels were associated with a higher risk of RKFD. Specifically, participants categorized in Class 2, Class 3, and Class 4 exhibited higher odds of experiencing RKFD compared to those in Class 1. In the fully adjusted model, the odds ratios (OR) for RKFD were 1.70 (95% confidence interval [CI]: 1.16–2.50) for Class 2, 1.48 (95% CI: 0.99-2.20) for Class 3, and 3.89 (95% CI: 2.70–5.66) for Class 4, with a significant trend (*P* for trend < 0.001). Additionally, cuCTI showed a positive association with RKFD, where each one-unit increment in cuCTI corresponded to an 18% increase in the risk of RKFD (OR = 1.18, 95% CI: 1.13–1.22, *P* < 0.001). When cuCTI was stratified into tertiles, the risk progressively escalated from the lowest to the highest tertile. In the fully adjusted model, individuals in the middle cuCTI tertile had an OR of 1.41 (95% CI: 0.98–2.05), while those in the highest tertile had an OR of 2.62 (95% CI: 1.87–3.72) compared to the lowest tertile, with a significant trend (*P* for trend < 0.001).

**TABLE 3:** Logistic regression results for the association of CTI control level and cuCTI with RKFD.

Variable	Model 1			Model 2			Model 3		
	OR	95% CI	*P-*value	OR	95% CI	*P-*value	OR	95% CI	*P-*value
CTI control levels									
Class 2 vs. Class 1	1.73	1.19, 2.53	0.004	1.70	1.16, 2.50	0.007	1.70	1.16, 2.50	0.007
Class 3 vs. Class 1	1.52	1.02, 2.25	0.038	1.48	0.99, 2.20	0.056	1.48	0.99, 2.20	0.056
Class 4 vs. Class 1	4.06	2.88, 5.79	< 0.001	3.89	2.70, 5.66	< 0.001	3.89	2.70, 5.66	< 0.001
*P* for trend			< 0.001			<0.001			< 0.001
Class 3 vs. Class 2	0.88	0.60, 1.27	0.487	0.87	0.60, 1.26	0.466	0.87	0.60, 1.26	0.466
CuCTI (continuous)	1.18	1.14, 1.22	< 0.001	1.18	1.13, 1.22	< 0.001	1.18	1.13, 1.22	< 0.001
Tertiles of cuCTI									
Q1	Reference			Reference				Reference	
Q2	1.47	1.02, 2.11	0.038	1.41	0.98, 2.05	0.063	1.41	0.98, 2.05	0.063
Q3	2.79	2.03, 3.91	< 0.001	2.62	1.87, 3.72	< 0.001	2.62	1.87, 3.72	< 0.001
*P* for trend			< 0.001			<0.001			< 0.001

Low cuCTI group[Q1], medium cuCTI group[Q2], high cuCTI group [Q3]; Significant *P* values < 0.05 are in bold. Model 1 was unadjusted. Model 2 was adjusted for age, gender, BMI and education. Model 3 was adjusted for age, gender, BMI, education, smoking status, drinking status, hypertension, diabetes, dyslipidemia, cardiovascular disease (including heart disease and stroke), chronic lung disease, liver disease, digestive disease, total cholesterol, HDL, LDL HbA1c, uric acid, and hemoglobin.

**FIGURE 2:**
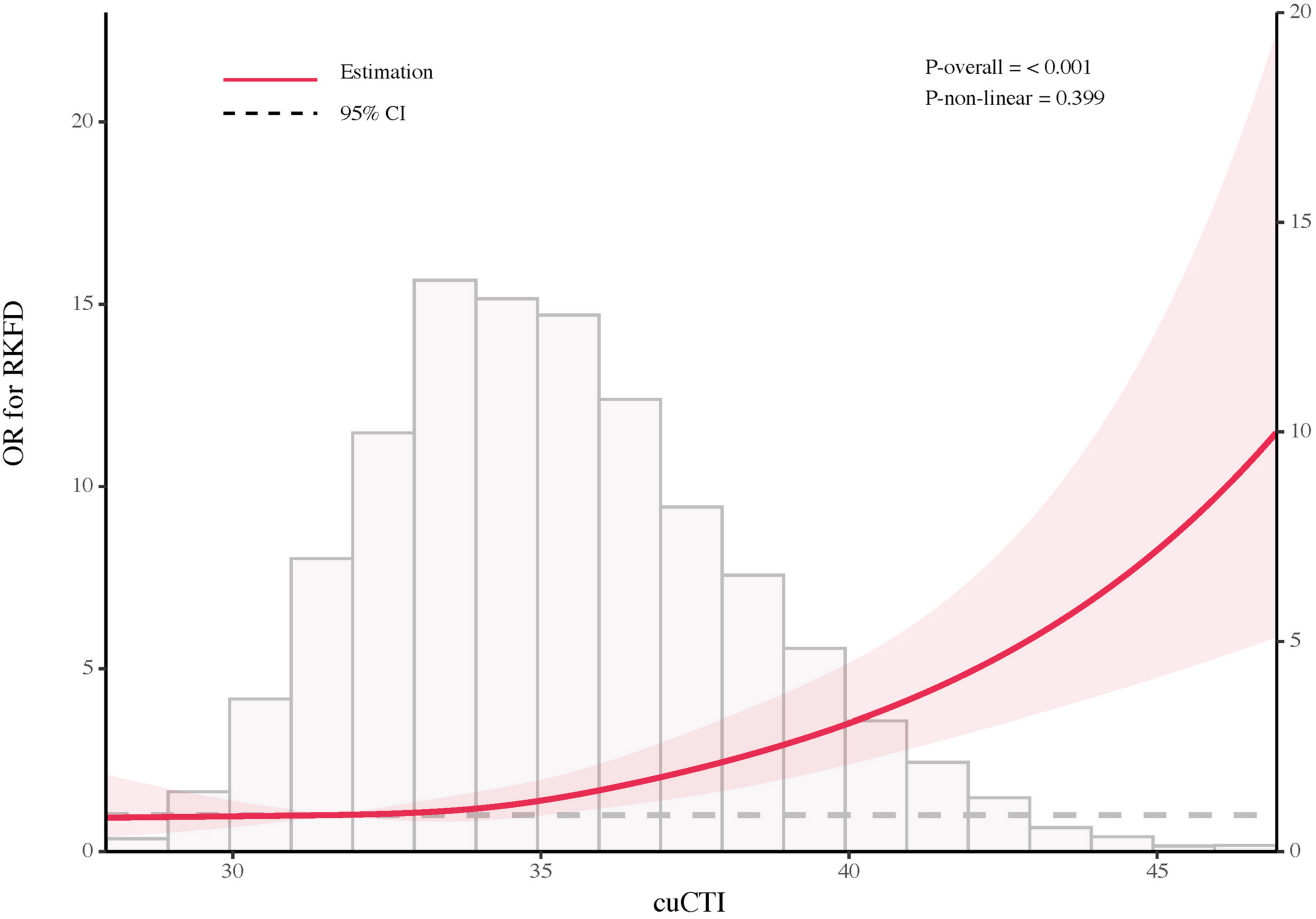
Restricted cubic spline curve showing the association between cuCTI and RKFD. Odds ratios (solid red line) and 95% confidence intervals (shaded area) were estimated from multivariable logistic regression models with restricted cubic splines. The histogram shows the distribution of cuCTI.

**FIGURE 3:**
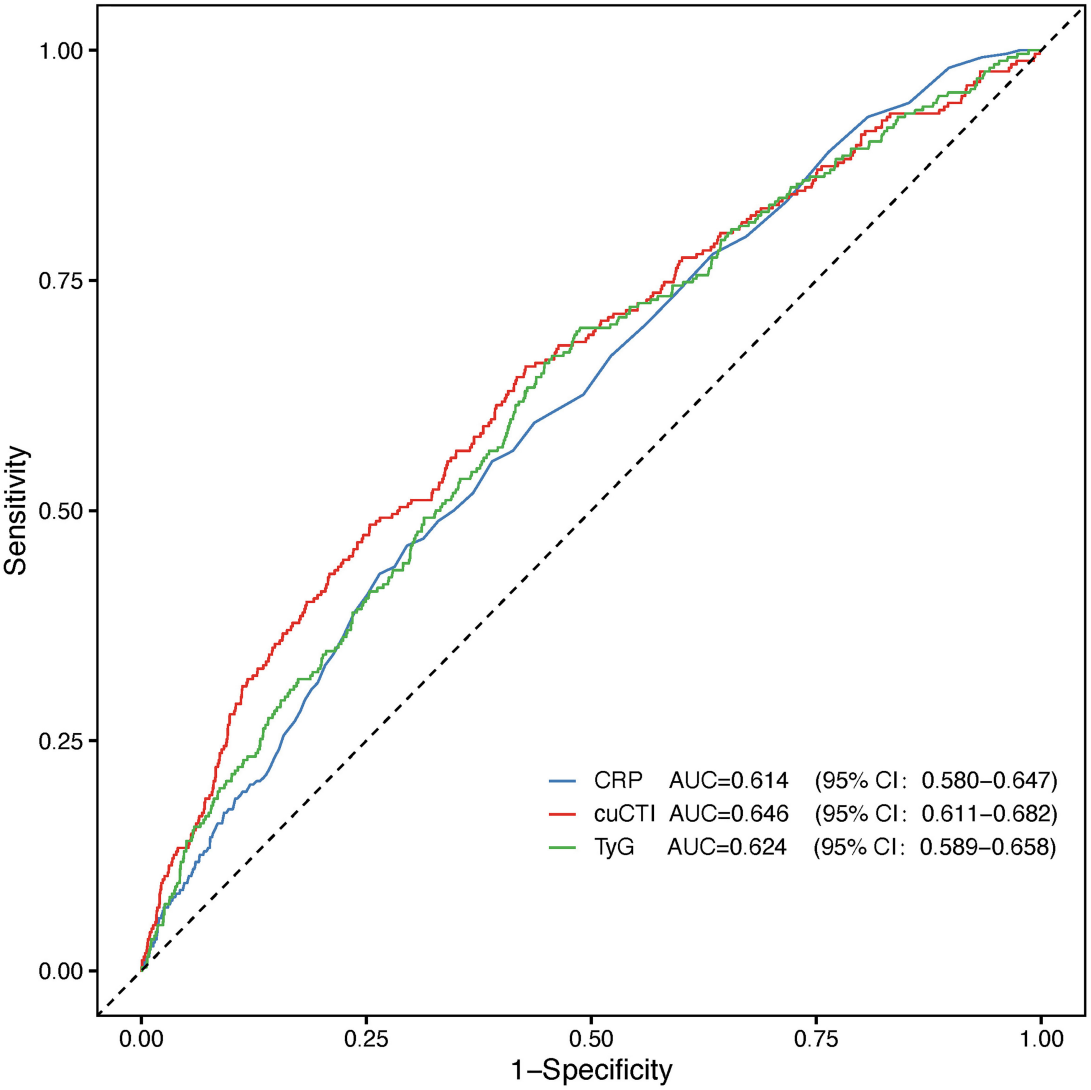
ROC curves for CRP, TyG, and cuCTI in predicting RKFD.

Multivariable spline regression models showed a linear rise in RKFD risk with increasing cuCTI (Figure 2). Individuals with lower cuCTI had a relatively low and stable risk. The increase in risk became steeper when cuCTI exceeded approximately 37. ROC curves were further constructed to compare the discriminatory performance of cuCTI, CRP, and TyG for RKFD, with cuCTI showing the largest AUC among the three indicators (Figure 3). Pairwise comparisons indicated statistical differences between cuCTI and CRP (*P* = 0.032) and between cuCTI and TyG (*P* = 0.045), whereas no difference was observed between CRP and TyG (*P* = 0.586).

### Subgroup analysis

**FIGURE 4:**
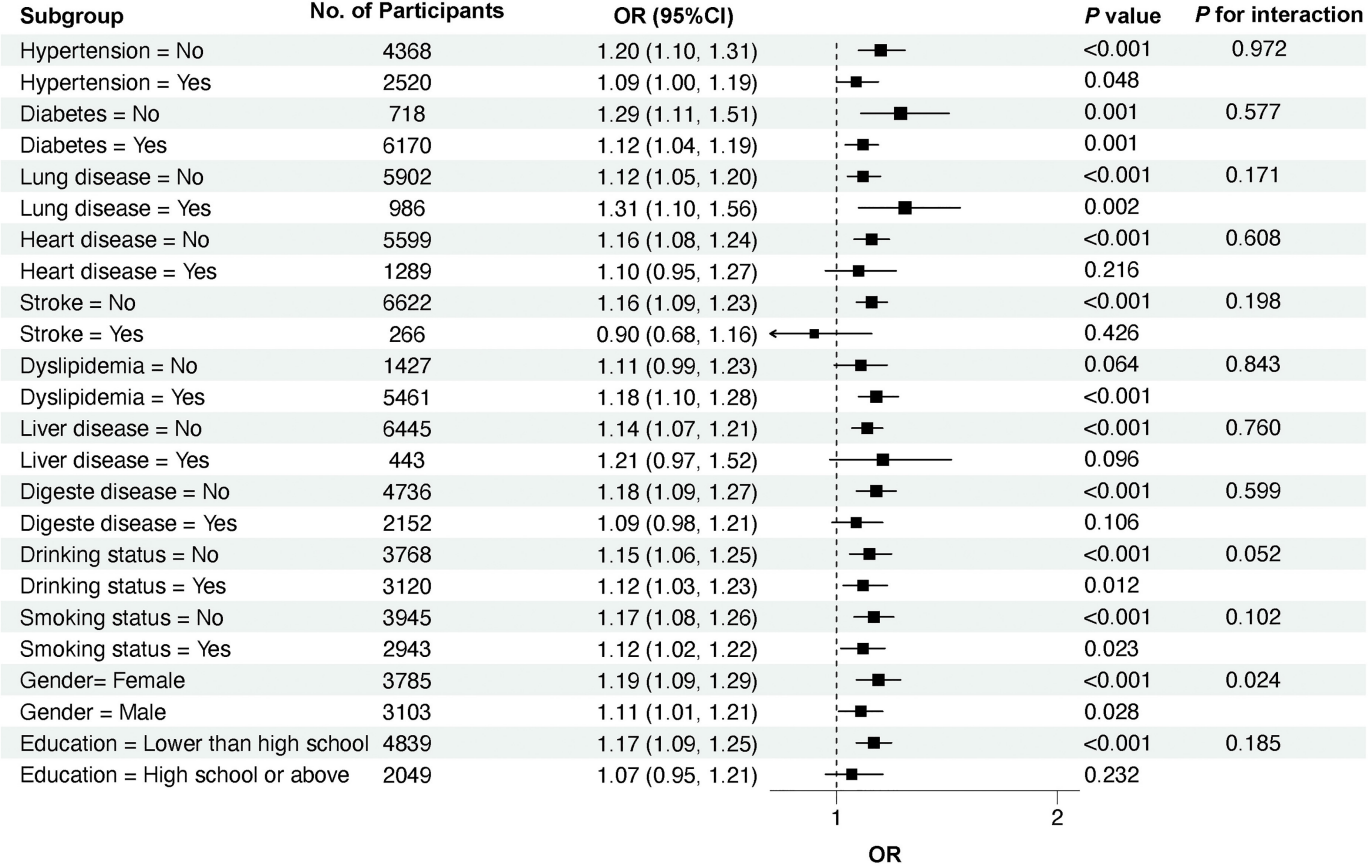
Subgroup analyses of the association between cuCTI and the risk of RKFD. This figure shows the link between cuCTI and kidney function decline in different subgroups. Each square shows the odds ratio (OR), and the line shows its 95% confidence interval. The dashed line marks OR = 1. The positive link between cuCTI and kidney decline was seen in most subgroups, and no clear interaction was found.

Figure 4 illustrates the subgroup analyses examining the association between cumulative changes in cuCTI and the risk of RKFD. A positive correlation was observed across nearly all subgroups. Individuals with larger cumulative increases in cuCTI were more likely to develop RKFD, regardless of age, sex, or baseline health status. While the magnitude of the effect exhibited slight variations, the overall direction remained consistent. No significant interaction was identified between cuCTI and other baseline variables.

Supplementary Table 1 corroborates these findings, demonstrating similar results when baseline CTI control levels were analyzed instead of cumulative changes. The trend remained consistent across all subgroups, with both higher cumulative increases and poorer control of CTI being associated with an elevated risk of RKFD.

### Sensitivity analyses

All datasets exhibited comparable odds ratios for RKFD, as presented in Supplementary Tables 2, 3. Regression analyses were performed separately within each imputed dataset, and the resulting estimates were combined using Rubin’s rules. The cuCTI and CTI control levels showed consistent effect estimates across the original dataset, each imputed dataset, and the pooled results. The findings remained robust across the original, imputed, and pooled datasets, with the associations maintaining consistency and remaining unaffected by data imputation. The OR estimates were slightly higher in the imputed datasets than in the original dataset. However, the differences were small, and the confidence intervals largely overlapped. These findings indicate that the multiple imputation procedure had little impact on the estimated association of cuCTI and RKFD.

### Reverse causality analysis

To assess potential reverse causality, we examined whether baseline renal function predicted subsequent changes in CTI using multivariable linear regression models. Baseline eGFR was not significantly associated with changes in CTI over time (β = 0.001, *P* = 0.435).

### External supportive analysis using NHANES

A total of 2015 participants aged ≥ 45 years from NHANES were included to further explore the association between CTI and kidney function. In weighted linear regression analyses, higher CTI levels were associated with lower eGFR after multivariable adjustment. When CTI was categorized into quartiles, participants in higher CTI categories tended to have lower eGFR compared with those in the lowest quartile, and a significant linear trend was observed across categories (Supplementary Table 4). Restricted cubic spline analysis showed a significant inverse association between CTI and eGFR, with no evidence of non-linearity (Supplementary Figure 2).

## Discussion

Chronic inflammation and metabolic imbalance are recognized contributors to kidney damage; however, there is a paucity of markers that effectively capture their long-term combined impact. Our findings indicate that elevated cuCTI levels were associated with a higher risk of RKFD in middle-aged and older adults, particularly among individuals with persistently high CTI levels. This association persisted even after adjusting for demographic, lifestyle, and clinical variables. Similar patterns were observed across various subgroups and sensitivity analyses. Participants with persistently high CTI levels showed the greatest risk, whereas those with increasing CTI levels also exhibited elevated risk compared with individuals with low and stable levels. In contrast to single-time assessments of metabolic or inflammatory markers, cuCTI quantifies both the magnitude and duration of exposure, thereby providing a more comprehensive perspective on chronic metabolic-inflammatory stress over an extended period.

The association between prolonged inflammatory-metabolic stress and the deterioration of kidney function likely involves multiple biological pathways. Chronic inflammation can increase glomerular vascular endothelial permeability, reduce nitric oxide activity, and promote fibrosis (32–34). These changes impair renal microcirculation and accelerate structural damage. Moreover, sustained inflammation can activate macrophages and fibroblasts, resulting in the accumulation of extracellular matrix and interstitial fibrosis in glomerular cells (35, 36). Metabolic dysfunction further exacerbates renal damage. Insulin resistance leads to aberrant glucose and lipid metabolism within renal tissues. Elevated triglyceride and glucose levels induce oxidative and endoplasmic reticulum stress, culminating in mitochondrial damage and tubular cell apoptosis (37, 38). Recent work has further emphasized that metabolic stress does not merely cause passive injury, but may drive metabolic reprograming in renal tubular cells. Under sustained lipid and glucose overload, tubular cells undergo shifts in substrate utilization, mitochondrial respiration, and fatty acid oxidation, with progressive loss of energetic flexibility and adaptive capacity (39). In this context, metabolic reprograming may rep resent a mechanistic bridge linking systemic metabolic–inflammatory stress to tubular vulnerability, maladaptive repair, and subsequent functional decline, rather than a simple downstream consequence of hyperglycemia or hypertriglyceridemia alone. Additionally, these metabolic stresses may activate the renin–angiotensin system, which in turn promotes local inflammation and fibrosis (40). This perspective may help explain why a composite index integrating inflammatory and metabolic signals can be more informative than isolated biochemical abnormalities when evaluating early renal risk.

Inflammation and metabolic stress are mutually reinforcing processes. Insulin resistance exacerbates inflammatory signaling, while inflammation exacerbates insulin resistance by disrupting adipokine balance and impairing insulin receptor function (41, 42). This bidirectional interaction establishes a chronic pro-inflammatory and pro-fibrotic milieu that progressively undermines renal resilience. Furthermore, compromised renal function can diminish the clearance of inflammatory mediators and metabolic byproducts, including uric acid, advanced glycation end-products, and lipid oxidation products. The accumulation of these substances further stimulates inflammation and oxidative stress, creating a self-perpetuating cycle that accelerates renal deterioration. CTI integrates markers of systemic inflammation (CRP) and metabolic dysfunction (triglycerides and fasting glucose), thereby reflecting the combined metabolic–inflammatory burden. Accordingly, elevated cuCTI may indicate prolonged exposure to these deleterious processes and serve as a marker of biological stress associated with rapid renal function decline.

The potential bidirectional relationship between renal dysfunction and metabolic–inflammatory stress warrants consideration. Early renal impairment may contribute to systemic inflammation and metabolic disturbances, partly due to reduced clearance of inflammatory mediators and metabolic byproducts. Consequently, subclinical renal dysfunction present at baseline may have partially influenced CTI levels. To further examine this possibility, we performed an inverse analysis to assess whether baseline renal function predicted subsequent changes in CTI. Baseline eGFR was not significantly associated with changes in CTI over time, indicating that baseline renal function was unlikely to be a major determinant of subsequent metabolic–inflammatory changes. In the present study, several design features were implemented to reduce the likelihood of reverse causation. We employed a prospective cohort design, excluded participants with CKD at baseline, and evaluated incident RKFD during follow-up. These methodological considerations strengthen the temporal relationship between CTI exposure and subsequent renal function decline. Nevertheless, a bidirectional interaction between renal function and metabolic–inflammatory status cannot be completely excluded.

Previous research has demonstrated that elevated levels of CRP are associated with reduced eGFR and an increased risk of chronic kidney disease (43, 44). Similar associations have been documented for insulin resistance and lipid disorders, both of which can disrupt renal hemodynamics and promote glomerular injury (8, 14, 45). These findings support the hypothesis that persistent inflammation and metabolic imbalance may coexist, collectively reflecting a prolonged stress state that jeopardizes renal health. Nevertheless, most prior studies have evaluated these markers independently or at a single time point. Single measurements of CRP or the TyG index are insufficient to fully capture the chronic and fluctuating nature of inflammatory–metabolic stress. Moreover, conventional renal biomarkers remain essential for evaluating current kidney function (46, 47), whereas CTI and cuCTI may be better viewed as complementary indicators reflecting systemic metabolic and inflammatory status. This distinction also helps clarify the incremental value of the present study. Although recent study had reported significant associations between CTI and CKD risk or renal decline in middle-aged and older Chinese populations, most were based on single-time-point CTI assessment (48). By contrast, our study focused on cumulative exposure by constructing cuCTI from repeated biomarker measurements, thereby capturing both the intensity and duration of metabolic–inflammatory burden over time. In addition, we incorporated CTI control patterns derived from repeated measurements to characterize temporal heterogeneity in exposure, and supplemented the longitudinal CHARLS findings with supportive analyses in NHANES. Accordingly, the main contribution of this study is not merely to confirm that CTI is associated with renal outcomes, but to show that cumulative metabolic–inflammatory burden may provide additional information beyond a single CTI measurement when evaluating the risk of rapid renal function decline. Consistent with this integrative concept, ROC analyses in the present study showed that cuCTI yielded the largest AUC among the three indicators. Although the improvement in AUC was modest, cuCTI integrates inflammatory and metabolic information and reflects cumulative exposure over time. The associations observed across various subgroups and sensitivity analyses suggest that this combined, time-integrated index may more accurately reflect the biological stress contributing to kidney vulnerability. Notably, the subgroup analysis revealed a significant interaction by hypertension status, suggesting that the strength of the association between CTI control levels and RKFD may vary according to baseline blood pressure status. A possible explanation for this heterogeneity is that hypertension is commonly accompanied by vascular dysfunction and renal microvascular injury, conditions that may alter the kidney’s response to persistent inflammatory–metabolic stress (49). Although causality cannot be definitively established, these findings further support the potential of chronic systemic inflammation and metabolic imbalance to jointly signal an early decline in renal function.

To further examine whether the observed associations could also be observed in an independent population, we conducted Supplementary analyses using data from the NHANES. In this nationally representative dataset, higher CTI levels were associated with lower eGFR in weighted regression models, and restricted cubic spline analysis revealed a non-linear decline in eGFR as CTI increased. Although the analysis of NHANES was cross-sectional and therefore differed from the longitudinal design of the CHARLS cohort, the overall direction and pattern of the associations were similar in the two populations. This consistency suggests that the link between metabolic–inflammatory burden and impaired kidney function may persist across different study designs and populations. Taken together, the NHANES results provide complementary evidence supporting the potential role of CTI as an indicator of metabolic–inflammatory stress related to early renal dysfunction.

The current study employed two complementary methodologies to characterize the long-term inflammatory and metabolic burden: the cumulative cuCTI and CTI control levels derived from K-means clustering. Both metrics were associated with the risk of accelerated kidney function decline, yet they encapsulate distinct dimensions of exposure. The CTI control levels represent the trajectory or pattern of inflammatory–metabolic status over time, differentiating individuals who maintain stable, low levels from those whose CTI increases, decreases, or remains persistently high. Although the increasing and decreasing CTI pattern groups showed opposite temporal trends, the difference in RKFD risk between these two groups was not statistically significant. These patterns are instrumental in identifying individuals experiencing unfavorable dynamic changes who may necessitate closer monitoring. Nevertheless, cluster classification is categorical and does not offer a continuous measure of cumulative stress. Conversely, the cuCTI quantifies the overall inflammatory–metabolic exposure by integrating both intensity and duration into a single continuous variable. It encapsulates long-term biological stress and provides a more direct estimation of the total burden on the kidneys. In this study, cuCTI demonstrated a linear relationship with the risk of accelerated kidney function decline, suggesting that even modest increases in cumulative exposure are associated with heightened renal vulnerability. The integration of CTI trajectories and cuCTI values provides a comprehensive understanding of the impact of chronic inflammation and metabolic processes on renal health. CTI control levels facilitate visualization of the evolution of individuals’ metabolic-inflammatory states, while cuCTI quantifies the cumulative effect of such exposure over time. Employing both methodologies enables clinicians and researchers to differentiate between short-term fluctuations and sustained long-term stress. Given that the components of CTI are routinely measured in clinical settings, these indices are applicable for large-scale health monitoring. Monitoring changes in CTI and cuCTI values may aid in the early identification of individuals at elevated risk for kidney function decline and inform preventive strategies aimed at maintaining long-term inflammatory and metabolic stability.

This study is subject to several limitations. Firstly, as an observational study, it does not establish causality between inflammation, metabolism, and renal decline. The cuCTI was assessed only twice, potentially overlooking short-term variations. Secondly, certain variables, including dietary habits, the use of medications that may influence CTI components (such as anti-inflammatory drugs, lipid-lowering agents, and glucose-lowering medications), and genetic factors, were not accounted for, partly because detailed and consistently available information on these factors was limited in the CHARLS dataset. Some lifestyle-related variables were not collected for all participants and were measured using relatively coarse self-reported survey items, which made them difficult to incorporate as reliable covariates in multivariable models. In addition, extensive imputation of variables with substantial missingness might itself introduce additional bias and uncertainty. Therefore, residual confounding due to unmeasured or incompletely measured variables cannot be fully excluded. This is an inherent limitation of observational studies and may have influenced the observed associations (50, 51). Finally, CRP was the sole inflammatory marker evaluated; incorporating a broader range of markers in future research could enhance the understanding of underlying biological mechanisms. Despite these limitations, this study contributes valuable insights from a national cohort, demonstrating that both cumulative and dynamic CTI can effectively characterize long-term metabolic and inflammatory stress. These findings may facilitate the early identification of individuals at elevated risk and support improved management of renal health.

## Conclusion

Our study identified an association between cumulative changes in the cuCTI and an elevated risk of RKFD, and the association was most pronounced among individuals with persistently high CTI levels. Long-term inflammatory and metabolic imbalances may serve as indicators of early RKFD in middle-aged and older adults.

## Data Availability

The raw data supporting the conclusions of this article will be made available by the author, without undue reservation.

## Footnotes

1
http://charls.pku.edu.cn/en/

## Supplementary material

Supplementary Figure 1 K-means clustering of CTI change patterns between 2011 and 2015. **(A)** The elbow plot shows the total within-cluster sum of squares for different numbers of clusters (k). The reduction in within-cluster variance became progressively smaller after *k* = 4, indicating diminishing gains with additional clusters. **(B)** Scatter plot showing the K-means clustering results based on CTI values measured in 2011 and 2015. Each color represents one of the four CTI pattern groups. **(C)** Median CTI values for each class are shown from 2011 to 2015. Class 1 had stable and low CTI levels. Class 2 showed an increasing trend. Class 3 showed a decreasing trend. Class 4 maintained the highest CTI levels over time.

Supplementary Figure 2 Restricted cubic spline analysis of the association between CTI and eGFR among NHANES participants. The solid red line represents the adjusted estimate, and the shaded area indicates the 95% confidence interval. The model was fully adjusted for demographic characteristics, lifestyle factors, cardiometabolic conditions, and metabolic biomarkers. A significant inverse association was observed (*P* < 0.001), with no evidence of non-linearity (*P* for non-linearity = 0.377).
