# Supplementary material for: Association between cumulative changes of the C-reactive protein-triglyceride glucose index and the incidence of rapid kidney function decline: a nationwide prospective cohort study
Source: Front Nutr. 2026 Apr 13;13:1795444. doi: 10.3389/fnut.2026.1795444 (PMC13111251; doi:10.3389/fnut.2026.1795444)
Supplement: Supplementary file 1 [file Table_1.docx]

| Table S1 Subgroup analysis of the association between CTI control levels and RKFD incidents | | | | | | | |
| --- | --- | --- | --- | --- | --- | --- | --- |
| Subgroup | No. of Participants | Class 1 | Class 2 | Class 3 | Class 4 | *P* value | *P* for interaction |
|  |  |  | OR (95% CI) | OR (95% CI) | OR (95% CI) |  |  |
| Hypertension |  |  |  |  |  |  | 0.016 |
| No | 4368 | Reference | 1.18 (0.70, 1.98) | 0.83 (0.47, 1.43) | 3.69 (1.96, 6.92) | 0.009 |  |
| Yes | 2520 | Reference | 2.16 (1.03, 5.00) | 2.29 (1.09, 5.28) | 2.66 (1.19, 6.47) | 0.048 |  |
| Diabetes |  |  |  |  |  |  | 0.539 |
| No | 718 | Reference | 1.83 (0.21, 39.24) | 2.87 (0.43, 57.50) | 10.70 (1.66, 214.57) | 0.002 |  |
| Yes | 6170 | Reference | 1.27 (0.84, 1.93) | 1.13 (0.73, 1.73) | 2.04 (1.23, 3.39) | 0.025 |  |
| Lung disease |  |  |  |  |  |  | 0.326 |
| No | 5902 | Reference | 1.38 (0.90, 2.14) | 1.34 (0.86, 2.08) | 2.42 (1.46, 4.02) | 0.002 |  |
| Yes | 986 | Reference | 0.82 (0.25, 2.67) | 0.42 (0.10, 1.56) | 2.54 (0.73, 9.02) | 0.243 |  |
| Heart disease |  |  |  |  |  |  | 0.578 |
| No | 5599 | Reference | 1.21 (0.77, 1.89) | 1.22 (0.78, 1.91) | 2.53 (1.52, 4.24) | 0.001 |  |
| Yes | 1289 | Reference | 2.04 (0.75, 6.14) | 1.13 (0.36, 3.64) | 2.55 (0.81, 8.68) | 0.352 |  |
| Dyslipidemia |  |  |  |  |  |  | 0.757 |
| No | 1427 | Reference | 0.80 (0.34, 1.91) | 0.91 (0.41, 2.12) | 1.60 (0.68, 3.94) | 0.137 |  |
| Yes | 5461 | Reference | 1.57 (0.99, 2.50) | 1.32 (0.81, 2.15) | 3.04 (1.71, 5.38) | 0.002 |  |
| Liver disease |  |  |  |  |  |  | 0.273 |
| No | 6445 | Reference | 1.22 (0.81, 1.86) | 1.08 (0.70, 1.66) | 2.34 (1.45, 3.79) | 0.003 |  |
| Yes | 443 | Reference | 6.22 (0.99, 55.91) | 4.83 (0.89, 39.44) | 7.56 (0.82, 88.64) | 0.118 |  |
| Digeste disease |  |  |  |  |  |  | 0.790 |
| No | 4736 | Reference | 1.23 (0.74, 2.07) | 1.18 (0.71, 1.97) | 2.74 (1.55, 4.88) | 0.002 |  |
| Yes | 2152 | Reference | 1.56 (0.81, 3.06) | 1.18 (0.57, 2.42) | 2.16 (0.96, 4.88) | 0.173 |  |
| Drinking status |  |  |  |  |  |  | 0.681 |
| No | 3768 | Reference | 1.12 (0.63, 2.02) | 1.18 (0.67, 2.11) | 2.11 (1.11, 4.06) | 0.025 |  |
| Yes | 3120 | Reference | 1.54 (0.87, 2.74) | 1.17 (0.63, 2.13) | 2.75 (1.38, 5.47) | 0.029 |  |
| Smoking status |  |  |  |  |  |  | 0.659 |
| No | 3945 | Reference | 1.33 (0.77, 2.29) | 1.27 (0.74, 2.20) | 2.64 (1.44, 4.90) | 0.004 |  |
| Yes | 2943 | Reference | 1.38 (0.74, 2.57) | 1.11 (0.58, 2.10) | 2.37 (1.14, 4.90) | 0.077 |  |
| Gender |  |  |  |  |  |  | 0.785 |
| Female | 3785 | Reference | 1.32 (0.74, 2.39) | 1.12 (0.62, 2.06) | 2.36 (1.23, 4.59) | 0.025 |  |
| Male | 3103 | Reference | 1.40 (0.79, 2.49) | 1.33 (0.75, 2.37) | 2.77 (1.40, 5.48) | 0.011 |  |
| Education |  |  |  |  |  |  | 0.475 |
| Lower than high school | 4839 | Reference | 1.32 (0.82, 2.14) | 1.12 (0.68, 1.86) | 2.77 (1.62, 4.77) | 0.001 |  |
| High school or above | 2049 | Reference | 1.31 (0.61, 2.86) | 1.35 (0.64, 2.85) | 1.66 (0.64, 4.26) | 0.307 |  |
| Note: Subgroup analyses were performed using Model 3, which was adjusted for age, sex, body mass index, education level, smoking status, alcohol consumption, hypertension, diabetes, dyslipidemia, cardiovascular disease (including heart disease and stroke), chronic lung disease, liver disease, digestive disease, total cholesterol, HDL-C, LDL-C, HbA1c, uric acid, and hemoglobin levels. The subgroup of participants with stroke was not presented in the class-based subgroup analysis because of the sparse distribution of stroke cases across the four CTI control level classes, which precluded reliable estimation within individual strata. In addition, some subgroup estimates had wide confidence intervals due to limited sample sizes within certain strata; therefore, these estimates should be interpreted with caution and primarily considered exploratory. | | | | | | | |
