# Supplementary material for: Association between cumulative changes of the C-reactive protein-triglyceride glucose index and the incidence of rapid kidney function decline: a nationwide prospective cohort study
Source: Front Nutr. 2026 Apr 13;13:1795444. doi: 10.3389/fnut.2026.1795444 (PMC13111251; doi:10.3389/fnut.2026.1795444)
Supplement: Supplementary file 2 [file Table_2.docx]

| Table S2. Association between cuCTI and RKFD in the original dataset and across multiple imputed datasets (Model 3). | | |
| --- | --- | --- |
| Dataset | OR (95% CI) | *P* value |
| Original | 1.12 (1.05–1.19) | <0.001 |
| Imputed #1 | 1.14 (1.08–1.21) | <0.001 |
| Imputed #2 | 1.15 (1.08–1.22) | <0.001 |
| Imputed #3 | 1.14 (1.08–1.21) | <0.001 |
| Imputed #4 | 1.14 (1.08–1.21) | <0.001 |
| Imputed #5 | 1.14 (1.08–1.21) | <0.001 |
| MI pooled (Rubin) | 1.14 (1.08–1.21) | <0.001 |
| **Note:** Odds ratios (ORs) were estimated using multivariable logistic regression (Model 3), with RKFD as the outcome. Results are presented for the original dataset, each of the five imputed datasets generated by multiple imputation, and the pooled estimates obtained using Rubin’s rules (MI pooled [Rubin]). *P* values represent the statistical significance of the association between cuCTI and RKFD within each dataset.  Model 3 was adjusted for age, gender, BMI, education, smoking status, drinking status, hypertension, diabetes, dyslipidemia, cardiovascular disease (including heart disease and stroke), chronic lung disease, liver disease, digestive disease, total cholesterol, HDL, LDL HbA1c, uric acid, and hemoglobin. | | |
