# Supplementary material for: Association between cumulative changes of the C-reactive protein-triglyceride glucose index and the incidence of rapid kidney function decline: a nationwide prospective cohort study
Source: Front Nutr. 2026 Apr 13;13:1795444. doi: 10.3389/fnut.2026.1795444 (PMC13111251; doi:10.3389/fnut.2026.1795444)
Supplement: Supplementary file 3 [file Table_3.docx]

| Table S3. Robustness of the association between CTI control levels (Class 1–4) and RKFD across the original and multiple imputed datasets (Model 3). | | | | | | | |
| --- | --- | --- | --- | --- | --- | --- | --- |
| Dataset | Class 1 | Class 2 | | Class 3 | | Class 4 | |
|  |  | OR (95% CI) | *P* value | OR (95% CI) | *P* value | OR (95% CI) | *P* value |
| Original | Reference | 1.29 (0.83–2.00) | 0.255 | 1.19 (0.76–1.86) | 0.435 | 2.25 (1.36–3.74) | 0.002 |
| Imputed #1 | Reference | 1.32 (0.88–1.99) | 0.176 | 1.21 (0.80–1.83) | 0.371 | 2.47 (1.56–3.94) | <0.001 |
| Imputed #2 | Reference | 1.32 (0.88–1.99) | 0.176 | 1.21 (0.80–1.83) | 0.361 | 2.48 (1.56–3.96) | <0.001 |
| Imputed #3 | Reference | 1.30 (0.87–1.96) | 0.197 | 1.19 (0.79–1.81) | 0.398 | 2.42 (1.53–3.86) | <0.001 |
| Imputed #4 | Reference | 1.32 (0.88–1.98) | 0.184 | 1.20 (0.79–1.82) | 0.382 | 2.46 (1.55–3.93) | <0.001 |
| Imputed #5 | Reference | 1.31 (0.88–1.97) | 0.191 | 1.20 (0.79–1.81) | 0.394 | 2.44 (1.54–3.90) | <0.001 |
| MI pooled (Rubin) | Reference | 1.70 (1.16, 2.50) | 0.185 | 1.48 (0.99, 2.20) | 0.381 | 3.89 (2.70, 5.66) | <0.001 |
| **Note:** Odds ratios (ORs) were estimated using multivariable logistic regression with RKFD as the outcome. Model 3 was adjusted for age, sex, body mass index, education level, smoking status, alcohol consumption, hypertension, diabetes, dyslipidemia, cardiovascular disease (including heart disease and stroke), chronic lung disease, liver disease, digestive disease, total cholesterol, HDL-C, LDL-C, HbA1c, uric acid, and hemoglobin levels. Results are presented for the original dataset, each of the five imputed datasets generated by multiple imputation, and the pooled estimates obtained using Rubin’s rules (MI pooled [Rubin]). P values represent the statistical significance of the association within each dataset. | | | | | | | |
