# Supplementary material for: Association between cumulative changes of the C-reactive protein-triglyceride glucose index and the incidence of rapid kidney function decline: a nationwide prospective cohort study
Source: Front Nutr. 2026 Apr 13;13:1795444. doi: 10.3389/fnut.2026.1795444 (PMC13111251; doi:10.3389/fnut.2026.1795444)
Supplement: Supplementary file 4 [file Table_4.docx]

| Table S4. Weighted linear regression analysis of the association between CTI and eGFR in NHANES participants | | | | | | |
| --- | --- | --- | --- | --- | --- | --- |
| Variable | Model 1 |  | Model 2 |  | Model 3 |  |
|  | β (95% CI) | *P* value | β (95% CI) | *P* value | β (95% CI) | *P* value |
| CTI (continuous) | -2.45 (-3.39, -1.51) | <0.001 | -0.86 (-1.59, -0.12) | 0.031 | -0.81 (-1.54, -0.08) | 0.040 |
| CTI quartiles |  |  |  |  |  |  |
| Q1 | Reference |  | Reference |  | Reference |  |
| Q2 | -5.06 (-7.38, -2.75) | <0.001 | -1.96 (-3.51, -0.40) | 0.022 | -1.84 (-3.43, -0.25) | 0.034 |
| Q3 | -4.43 (-6.41, -2.46) | <0.001 | -2.10 (-3.81, -0.39) | 0.010 | -2.05 (-3.76, -0.34) | 0.016 |
| Q4 | -5.35 (-7.52, -3.17) | <0.001 | -2.44 (-4.14, -0.75) | 0.025 | -2.24 (-3.91, -0.58) | 0.029 |
| *P* for trend |  | <0.001 |  | 0.014 |  | 0.020 |
| Model 1 was unadjusted. Model 2 was adjusted for age, sex, and race. Model 3 was adjusted for age, sex, race, marital status, alcohol consumption, smoking status, body mass index, hypertension, diabetes. CTI quartiles were defined based on the weighted distribution of the study population. P for trend was calculated by including the ordinal CTI quartile variable as a continuous term in the regression model. All analyses incorporated NHANES sampling weights, strata, and primary sampling units. | | | | | | |
