# Supplementary figures and images for: Association between cumulative changes of the C-reactive protein-triglyceride glucose index and the incidence of rapid kidney function decline: a nationwide prospective cohort study
Source: Front Nutr. 2026 Apr 13;13:1795444. doi: 10.3389/fnut.2026.1795444 (PMC13111251; doi:10.3389/fnut.2026.1795444)

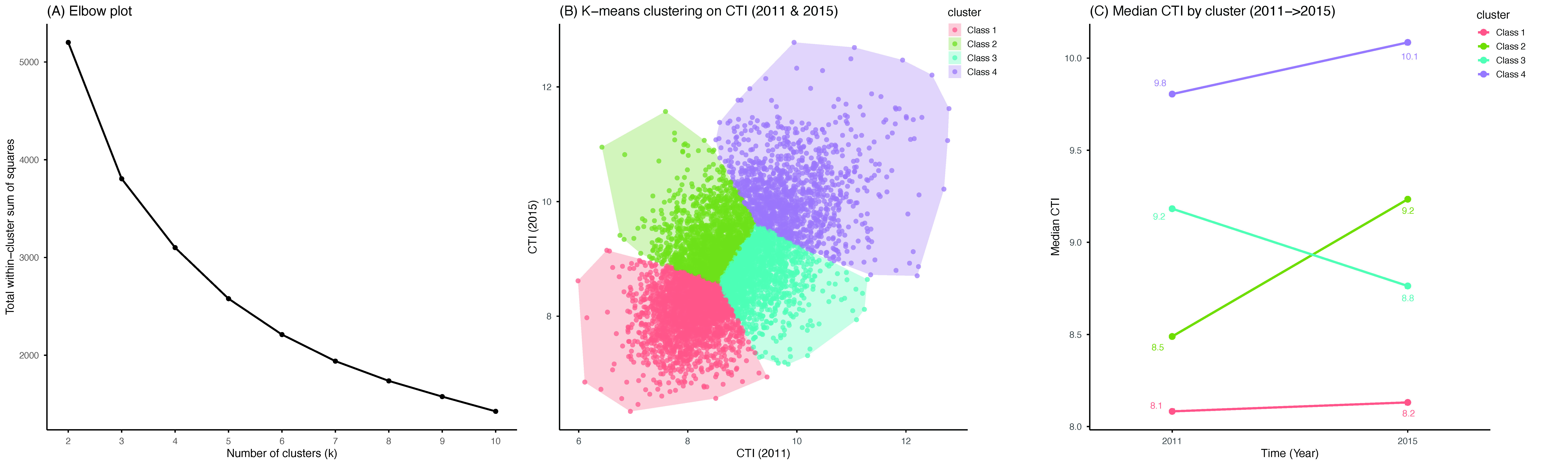

Supplement: Supplementary Figure 1 — K-means clustering of CTI change patterns between 2011 and 2015. (A) The elbow plot shows the total within-cluster sum of squares for different numbers of clusters (k). The reduction in within-cluster variance became progressively smaller after k = 4, indicating diminishing gains with additional clusters. (B) Scatter plot showing the K-means clustering results based on CTI values measured in 2011 and 2015. Each color represents one of the four CTI pattern groups. (C) Median CTI values for each class are shown from 2011 to 2015. Class 1 had stable and low CTI levels. Class 2 showed an increasing trend. Class 3 showed a decreasing trend. Class 4 maintained the highest CTI levels over time. [file Image_1.jpeg]

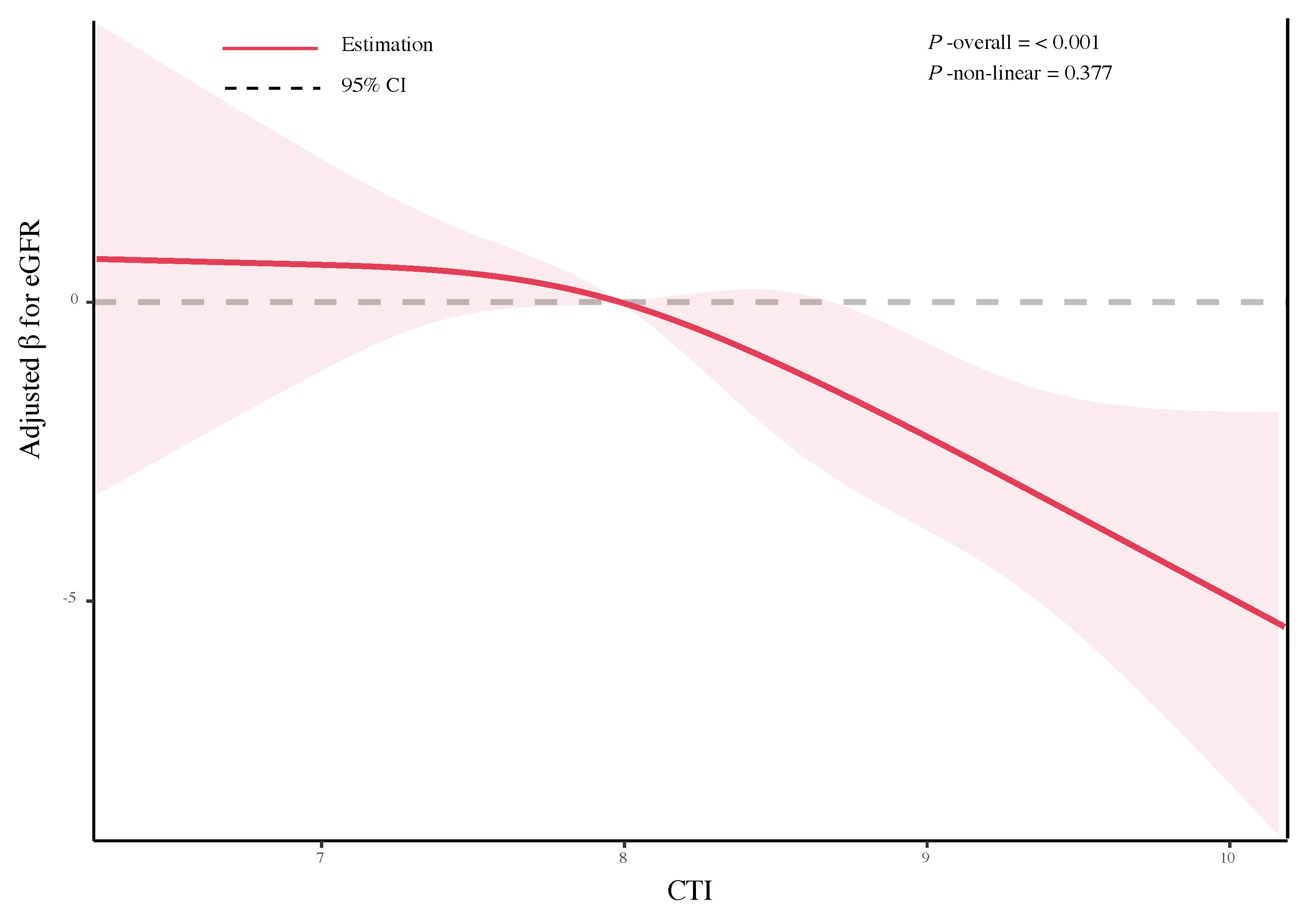

Supplement: Supplementary Figure 2 — Restricted cubic spline analysis of the association between CTI and eGFR among NHANES participants. The solid red line represents the adjusted estimate, and the shaded area indicates the 95% confidence interval. The model was fully adjusted for demographic characteristics, lifestyle factors, cardiometabolic conditions, and metabolic biomarkers. A significant inverse association was observed (P < 0.001), with no evidence of non-linearity (P for non-linearity = 0.377). [file Image_2.jpeg]
